# “I don't mean to be rude, but could you put a mask on while I'm here?” A qualitative study of risks experienced by domiciliary care workers in Wales during the COVID‐19 pandemic

**DOI:** 10.1111/hsc.14109

**Published:** 2022-11-24

**Authors:** Hayley Prout, Fiona V. Lugg‐Widger, Lucy Brookes‐Howell, Rebecca Cannings‐John, Ashley Akbari, Ann John, Daniel Rh. Thomas, Michael Robling

**Affiliations:** ^1^ Centre for Trials Research, Heath Park Campus Cardiff University Cardiff UK; ^2^ Population Data Science, Health Data Research UK (HDR UK) Swansea University Swansea UK; ^3^ Population Data Science, Administrative Data Research Wales Swansea University Swansea UK; ^4^ Public Health Wales, Communicable Disease Surveillance Centre Cardiff UK; ^5^ Cardiff Metropolitan University School of Health Sciences Cardiff UK; ^6^ DECIPHer – Centre for Development, Evaluation, Complexity and Implementation in Public Health Improvement Cardiff University Cardiff UK

**Keywords:** COVID‐19, domiciliary care workers, qualitative, risk, social care

## Abstract

Domiciliary care workers (DCWs) continued to provide care to adults in their own homes throughout the COVID‐19 pandemic. The evidence of the impact of COVID‐19 on health outcomes of DCWs is currently mixed. The OSCAR study will quantify the impact of COVID‐19 upon health outcomes of DCWs in Wales, explore causes of variation and extrapolate to the rest of the UK DCW population. An embedded qualitative study aimed to explore DCW experiences during the pandemic, including factors that may have varied risk of exposure to COVID‐19 and adverse health and wellbeing outcomes. Registered DCWs working throughout Wales were invited to participate in a semi‐structured telephone interview. 24 DCWs were interviewed between February and July 2021. Themes were identified through inductive analysis using thematic coding. Several themes emerged relating to risk of exposure to COVID‐19. First, general changes to the role of the DCW during the pandemic were identified. Second, practical challenges for DCWs in the workplace were reported, including staff shortages, clients and families not following safety procedures, initial shortages of personal protective equipment (PPE), DCW criticism of standard use PPE, client difficulty with PPE and management of rapid antigen testing. Third, lack of government/employer preparation for a pandemic was described, including the reorganisation of staff clients and services, inadequate or confusing information for many DCWs, COVID‐19 training and the need for improved practical instruction and limited official standard risk assessments for DCWs. Pressure to attend work and perceptions of COVID‐19 risk and vaccination was also reported. In summary, this paper describes the risk factors associated with working during the pandemic. We have mapped recommendations for each problem using these qualitative findings including tailored training and better support for isolated team members and identified the required changes at several socio‐ecological levels.

**What is known about this topic**

- Domiciliary care workers provided care in the community throughout the COVID‐19 pandemic
- Evidence to date is mixed regarding the impact of the pandemic on these workers
- Specific risks for care workers posed by working during the pandemic remain under‐explored.

**What this paper adds**

- Team working and care workers' roles changed during the pandemic identifying a training gap for those working in different ways and those redeployed.
- Factors contributing to risk to DCWs health and wellbeing included practical challenges such as shortages of staff and PPE and clients' understanding/following protective measures.
- Lack of preparedness resulted in inadequate and confusing information and training not meeting care worker needs.

## 1 BACKGROUND

The adult social care sector employed 1.5 million people across 17,700 organisations in England (2019), including 685,000 staff as domiciliary care workers (DCWs; Hayes et al., 2020; Skills for Care, 2021). In Wales, where this study took place, nearly 22,000 people work as DCWs (Social Care Wales, 2021a, 2021b) and all are registered with Social Care Wales (a Welsh‐Government‐sponsored body). DCWs in Wales provide a wide range of support including preventative services, support for independent living and personal care (Social Care Wales [SCW], 2018). These activities require close contact with clients, who may be at high risk from COVID‐19, with some within the UK Government's ‘clinically extremely vulnerable' category (Hayes et al., 2020).

Limited and mixed evidence exists on the impact of COVID‐19 on this crucial workforce. The Office for National Statistics (ONS) reported mortality rates for ‘Care workers and home carers' (SOC2010 code 6145) of 71.1 (males) and 25.9 (females) per 100,000 between 9 March and 25 May 2020 as compared with rates in the general population of 19.1 (males) and 9.7 (females) per 100,000. It is important to note that the occupational classification was absent in many ONS records (17.5% and 37.5% missing for males and females respectively; ONS, 2020). In addition, the impact on other health and well‐being outcomes were not reported (or indeed collected). Rates of testing positive for COVID‐19 (via a Polymerase Chain Reaction [PCR] test) in June 2020 found rates of infection amongst the DCW workforce to be similar to that of the general population (Public Health England, 2020).

The OSCAR study (Outcomes for Social Carers: an Analysis using Routine data)~is using a mixed‐methods design to quantify the impact of COVID‐19 upon health outcomes of DCWs in Wales, to explore causes of variation, and to extrapolate to the rest of the UK DCW population (Lugg‐Widger et al., 2021). Through data provided on the DCW workforce combined with electronic health record (EHR) outcome data within the Secure Anonymised Information Linkage (SAIL) Databank, the study will be the first to comprehensively quantify the risk of COVID‐19 on the UK DCW workforce. This paper describes the (OSCAR) study's qualitative component, which aimed to explore DCW experiences during the pandemic, including factors that may have varied risk of exposure to COVID‐19 and adverse health and wellbeing outcomes. The paper reports on risks associated with DCWs working through the pandemic.

## 2 METHODS

### 2.1 Study design

Within a mixed‐methods study, in‐depth qualitative telephone interviews were conducted to explore DCW pandemic working experiences and inform anonymised individual‐level population‐scale routine data linkage (Lugg‐Widger et al., 2021).

### 2.2 Eligibility

Participants were eligible if they were registered or working towards registration as a DCW with Social Care Wales (SCW), had actively worked for at least 4 months during one or both initial waves of the COVID‐19 pandemic in Wales and were able to provide consent.

### 2.3 Identification and approach

The proposed sample size for the qualitative study was 30. DCWs were informed and invited to participate through networks such as care‐providing organisations, unions and Social Care Wales. Informal methods of contacting DCWs included websites, social media (Twitter) and ‘snowballing’ where participants informed work colleagues of the study. Recruitment and interviews were carried out between February and July 2021. Interested DCWs contacted the qualitative researcher and had the opportunity to discuss any questions they had about the study. DCWs were offered a £20 Amazon voucher to participate. On agreement to interview, the researcher requested basic information to establish eligibility and ensure maximum variation in the sample. Screening questions included the number of years working as DCW, sex, ethnicity, region of work and employer classification. DCWs were immediately notified following screening if the interview could proceed and a suitable time was agreed.

### 2.4 The qualitative interviews and informed consent

Before the interview commenced, the researcher took informed consent remotely via telephone using Skype for Business and recorded the discussion using a research ethics committee (REC) approved consent script (Appendix S2.). The audio‐recorded interviews were carried out remotely, with DCWs given the option of using either telephone or online video recording software. The interviews were carried out at a place of choice for the DCWs, (e.g. the office, at home). The interviews were conducted by a clinically trained female, experienced researcher (HP), although her clinical background was not disclosed. The interview guide topics included DCWs' backgrounds, experiences of working practices before and during the COVID‐19 pandemic, feelings about how the COVID‐19 pandemic affected DCWs personally and an exploration of vaccinations, including the COVID‐19 vaccinations. Participants were also given the opportunity to discuss any other topic that they considered relevant. The interviewer made notes and regularly discussed anonymised content with the other qualitative researcher and study team. The interviewer grouped together similar content to make a list of initial codes, when interviewees were no longer raising or responding with new content that could not fit into main list of codes it was deemed that no more interviews were needed and data saturation had been reached.

### 2.5 Qualitative data analysis

Audio recordings were transcribed verbatim, anonymised and uploaded to NVivo 12 qualitative analysis software (QSR International, 2018). The researcher coded and developed themes from the data using inductive thematic analysis (Braun & Clarke, 2006). Thematic analysis used the six‐step process to analyse data as per Braun and Clarke (2006).

## 3 RESULTS

Recruitment: Following initial contact with the researcher, six DCWs did not reply to the researcher following receipt of the study information letter. Twenty‐four DCWs were recruited and interviewed (Table 1), with data saturation being reached at this point. The interviews lasted between 28 and 76 min (mean duration of 50 min).

**TABLE 1:** Interviewee demographics

Role type^a^	12 Care workers; 12 support workers
In an advanced role^b^	Seven of the care workers; six of the support workers
Sex	21 Female; 3 Male
Ethnicity	22 White (Welsh/English/British/Other) 2 Black/African/Caribbean/Black British/Other
Age	23–60 years (average of 45 years)
Region of employment	13 South Wales; 6 North Wales; 4 Mid Wales; 1 West Wales
Years as a DCW	6 months—21 years (average of 8 years)

^a^ Those visiting several client homes are referred to as Care Workers; those working in single supported living homes, home to several clients, are referred to as Support Workers.

^b^ Advanced role refers to Team Leader, Care Coordinator or Management position as reported by participant during the interview.

Characteristics of the participants: Although all interviewed individuals were registered with SCW as DCWs (SCW, 2021b), they comprised two groups; those that visited several client homes (referred to here as Care Workers [CWs]) and those that worked mostly in single supported living homes which were home to several clients (referred to here as Support Workers [SWs]). Participants reported working for a mixture of private, local authority and/or charity or via contracts through the local authority delivered by private or charity organisations.

Five themes are described in this paper related to the risk of exposure to COVID‐19 to DCWs: changes to DCW roles, practical workplace challenges, lack of Government/Company preparation for the pandemic, pressures to work and perceptions of COVID‐19 risk and of vaccinations. Quotes referenced throughout are in Boxes 1, 2, 3, 4.

BOX 1 **Practical challenges for DCWs in the workplace during the pandemic**

**Table:** 

*Staff shortages*	
Clients still requiring care in spite of the pandemic	‘Everyone felt quite the same, you know, we don't really want to be putting ourselves in that situation, but we understand we've got to, because there's no–one else there to do it. We have to, that's our job. The care doesn't stop because they've got Covid' (id 2 CW) ‘I never once thought you know, I can't be in work, because I wanted to be there for the people I support, because they needed, you know, me and my colleagues more than ever you know.’ (id 16 SW)
Clients still requiring care in spite of a reduced workforce	‘so suddenly, suddenly you know, you've got five staff members who're experiencing symptoms and they go off and you need to cover their shifts…. It was disastrous.’ (id 1 CW)
Waiting for results of PCR tests causing lengthier staff absences	‘so that's at least 72 hours we need to cover their work’ and if the test is positive, the staff member has ‘to stay off for 10 to 14 days’ (id 2 CW)
Fear of catching COVID‐19 possibly causing reduced applications for DCWs	‘I think because people are scared because of the pandemic so that has definitely had a massive impact in recruiting new staff’ (id 1 CW) ‘it's recruitment, um it just seems really, really difficult to, to retain and, and employ staff, that are wanting to work frontline’. (id 13 CW)
New DCW recruits not suitable for the role	‘we're finding that we are employing some people and their just heart, their heart isn't in it, they just want a job and it's like ….you know you've got a job, but then caring is another step up, you know you need to have those personal qualities … you know your empathy and everything’ (id 5 CW)
Media coverage not helping recruitment	‘throughout COVID, I don't believe or I don't feel that kind of care homes, nursing homes or anybody in the care industry, had the best of media coverage. … because you were continually hearing oh, well, care homes are this, and now it's spreading like wildfire in care homes, you know, when actually’ (id 24 SW)
Lack of childcare leading to staff shortages	‘some parents are needing the time off to look after children, because they're at home all day and there's no other childcare provision…… Um, you have to be a keyworker to, to access the childcare provisions in school’. (id 13 CW)
*Clients/families and private workers not following safety procedures*	
Families not wearing face masks, a particular problem	‘But it is mostly the families bringing it in, you see, and not wearing their masks. And we've had a couple, we had one little bit of an issue with one client, um, because, bless her, she's got six kids which all come in and out and visit her. Um, some do her shopping and things like that, um, ….again, all coming in not wearing masks’. (id 2 CW)
Clients did not follow safety precautions	[the clients] ‘just could not get the hang of it’ (id 7 CW)
Private carers not wearing face masks	‘So, I do find that they're complacent, and a couple of times I went into the one, the husband and wife and I went in and the private carer was in and she didn't have a mask on and I did have to say to her “Look, I don't mean to be rude, but could you put a mask on while I'm here?”’ (id 23 CW)
*Initial Shortages of PPE*	
Shortages of PPE	‘Because it was an absolute nightmare getting PPE from your suppliers and of course, suddenly their prices shot up because everyone wanted PPE’ (id 1 CW)
*DCW criticism of standard issue PPE*	
Deficiencies of standard issue face masks	‘whereas the ones that we were given free from the company …kept steaming up and steaming up, and I think they spent more time playing and cleaning these visors, that that's how they, I think that's how they got infected’ (id 21 SW) ‘They don't really work, so you've got to lift it up to try to keep … you can't be cleaning someone's teeth, or trying to bath them, if you can't see what you're doing. You need to see’ (id 14 SW)
Preference for alternative face masks	‘I was here in the thick of it, working every day that week because everybody was going down one by one … and I was the only one that didn't get it. … But I had already prepared. I had bought myself this [mask]. I came in here and I did not take that off' (id 21 SW)
Time consuming to put on PPE	‘it's definitely been more time consuming because of putting PPE on and donning and doffing, taking it off at the right times’ (id 24 SW)
*Client difficulties with DCWs wearing PPE*	
Clients feeling intimidated by PPE	‘we've got a couple of clients, who have got Alzheimer's, you know, they're at the point now, where they're really late stages of it, who are in bed and can't talk and, you know, I think it's a bit intimidating for them, when two of us come in, all masked up and PPE'd up and they, they kind of look at us, as if to say, oh what's happened here’ (id 3 CW)
Face mask displacement to aid recognition	‘you just pull it [the mask] down a little bit, so they [the clients] can see your mouth, and then you smile for them, and then you pull it back up….so when you're communicating you have to stand right back'. (id 8 CW)
Client dislike of PPE	‘oh, can't you take all that rubbish off’ (id 23 CW)
*Management of lateral flow testing: positive reports but some queries*	
Proof of lack of infection advantageous	‘I think it's a good thing to have in place, to um, you know, minimise the risk of bringing COVID into the house, because we ask people to test um, at least you know, the day before that they're coming on shift’ (id 16 SW) ‘it gives me peace of mind’ (id 22 SW) ‘When I do my test in the morning, and then if I go and visit my dad, I know I'm alright’ (id 23 CW) Er, I think it's brilliant, if um … because there was one lady who didn't have any symptoms whatsoever, and she was positive…. I think it's good…anyone that's going into someone else's home I think should have the test’ (id 8 CW)
DCWs received tests too late.	‘I'm pretty disgusted by it actually. … because I was begging and pleading … can we please have tests, that's all the staff was asking for, and myself, was tests, because it would have put our, our minds at ease’ (id 24 SW)
Queried efficacy of tests	‘They're not proven to have the greatest accuracy um I believe’ (id 12 SW)
Tests associated with false positive results	‘I have heard that um they're not very accurate and they can provide false, false positives apparently’ (id 16 SW)

BOX 2 **Lack of governmental/company preparation for a pandemic**

**Table:** 

*Reorganisation of staff, clients and services*	
Initial panic due to reorganisations	‘Er, well I would say in the beginning, um, quite frankly, chaos’ (id 15 SW) ‘So, it was quite manic at the start going back to last March’ (id 11 CW) ‘There was one … one [respite] bed in the county available, and that was for emergencies, and I think there were either one or two people there, and they would have kept an emergency bed as well’ (id 15 SW) ‘It was just absolutely mad’ (id 22 SW)
Transitioning from a support worker role to a care worker role	‘from my point of view of trying to learn a new job, erm, and trying to learn everybody's needs and where they lived and … like you know medication and everything … it was very stressful, it felt, I mean I did feel physically ill, erm … after the shifts because I hadn't had enough to eat. I mean I felt really shaky and … dehydrated and sick you know’ (id 9 CW)
*COVID‐19 training and the need for improved practical instruction*	
More specific training required	‘maybe it could be, maybe a little bit more specific, to you know, workplace…. it was enough information and obviously, we had you know, information from, other resources, but, maybe just, maybe a bit more specific’ (id 16 SW)

BOX 3 **Pressure to attend work during the pandemic**

**Table:** 

DCWs thankful for the statutory sick pay enhancement scheme	‘there was a change to like the Statutory Sick Pay and the Government would, um make up the shortfall for if you had to be off isolating….which I think put a lot of people's minds at ease’ (id 12 SW)
Statutory sickness payment too low	‘in the beginning yeah, you were just thinking oh my god, how're you going to pay everything if you're only on statutory sick pay it?’ (id 6 SW)
Financial pressure to attend work if ill	*‘*I was panicking a bit, if it did happen, but any situation, obviously, because you've still got to pay your rent and your car and all those sorts of things’ (id 3 CW) ‘we've got bills to pay haven't we’ (id 8 CW)
Potential dishonesty of reporting COVID‐19 symptoms to avoid losing pay	‘I know there are people that work in places that have gone to work, potentially, with COVID, because they just can't afford to be off’ (id 23 CW) ‘the minimum wage, how many people that might be minimum wage, can afford to have time off? … it's a conversation that came up once … how many people might be stuck, how many people might tell the truth’? (SW 14) ‘usually what you find with staff is, erm, that they can't afford to take that deduction, so they'll come back to work as soon as they can’ (id 17 SW)
Perceived immorality of losing pay for reporting a positive COVID‐19 test	‘for doing the right thing, that you lose your money for your shift, is just like, it's actually just really morally wrong isn't it, you know’? (id 15 SW)
Feeling of guilt for taking sick leave	‘I felt absolutely terrible because being in the job for this long I know the pressure the office staff fall under trying to cover all the shifts, erm but I couldn't do anything’ (id 1 CW)
The pressure to work weekends	‘because it's this kind of like unwritten rule, you really don't go sick on the weekend, if it's your weekend of working, you've got to work it. So, I worked the weekend’ (id 23 CW)
Belief that some DCWs were being dishonest about having COVID‐19 symptoms	‘Some tried to pull out the covid care, I need to go and have a test, oh, my test isn't until tomorrow so then they would try and pull a fast one to try and get some time off work' (id 5 CW)

BOX 4 **Perceptions of Covid‐19 and vaccinations**

**Table:** 

*Varied perceptions of becoming infected with the Covid‐19 virus*	
Perception of security following COVID‐19 vaccinations and that it halts transmission	‘I just feel a bit more protected. Obviously you've still got to think about you passing it on, but I just feel a bit invincible’ (id 8 CW) ‘I've already had the jabs. I'm probably one of the safest persons to be around now because I've had Covid and I got tested for the antibodies. I had the test for the antibodies and I've had the jab now’ (id 1 CW)
Proving vaccination status is no different to driving licence or travel passport	‘we have to have ID for driving licence, we have to have a passport, if we want to go abroad, I don't see what the problem is, with having some, sort of, validation, to say that we've been vaccinated’ (id 18 SW)
Trust placed in vaccinations	‘There's obviously a reason why, people recommend them [the vaccinations] ….they're not just doing it for the sake of it, so … no, I'm all for vaccinations’.(id 5 CW) ‘I was very pleased to be offered my Covid jab and also for my clients’ (id 7 CW)
Proposed origins of the COVID‐19 virus	‘Erm, I was watching somebody from the World Health Organisation the other day, and they've been over to China, haven't they, because of the rumours that it might be … that it came from there originally. And, that's, erm, well some people were saying that it's been released from a lab and things like that, and they've stated, no it didn't come from a lab. And what they've got is, it's been passed on to an animal, who has passed it on to a human' (id 19 SW)
The vaccination was designed by the Chinese Government to kill older people	‘did this on purpose and they're just trying to kill off the old people’ (id 23 CW)
The COVID‐19 virus is more serious than influenza	‘I was very paranoid when my daughter was young, when bird flu was going around and I made sure both of us got it then … [but] COVID's a different kettle of fish' (id 9 CW)
*Varied perceptions of the efficacy of the Covid‐19 vaccination*	
Lack of information regarding the COVID‐19 vaccination	[I wanted to know] ‘how it would affect me as a person really’ (id 24 SW) ‘we knew nothing about it did we, you know, it was all kind of the vaccines are coming out now, okay, front line workers are going to be in group one along with extremely vulnerable people’ (id 24 SW)
Pressure to have the COVID‐19 vaccination	‘It's up to you whether you have it or not, we can't force you, it's not mandatory ….Um, obviously, we'd like you to have it… (id 23 CW) ‘all of a sudden, we had a phone call, going, right, you're going this afternoon’ (id 23 CW) I thought well, I, I've got to have it done, you know what I mean’ (id 23 CW)
Queries whether natural immunity is better than vaccination	[is it] ‘better to fight the infection naturally or to have a bit of protection from the vaccination’ (id 9 CW)
Queries regarding composition of the vaccinations	‘I never quite trust, erm, the ingredients in them, they always say there's chemicals … heavy metals or something in them, mercury or something (id 12 SW)
Fear that the vaccination may not be safe	‘they were saying, right, well we're going to invent this vaccine, and I was thinking, well, well it's all very well saying you're going to invent it, how do we know a, that it's going to work and b, that you're not going to kill us all off, you know?’ (id 23 CW)
Lack of long term studies regarding the vaccine safety profile	‘they are trying their best to assure people that it's gone through the same tests as the flu jab. But because this has been going on for just a year, people are very sceptical …very, very sceptical’ (id 2 CW) ‘But they don't really know what the long‐term effects are. You've also got, erm, people with blood clots at the moment. It's only a tiny, tiny amount of people that's got it, and … but there's this been this panic, hasn't there, about people getting them’ (id 19 SW) Fertility issues concerning in view of the ‘short span of time it's taken them to make the vaccine’. (id 2 CW)
Futility of vaccinations in view of the virus mutating	‘like Covid is, it's a virus that mutates, you never get the same injections, do you, because they mutate… but this is going to be a situation where you have to have the injection because it mutates, you've seen that all over the world, you know, getting their own versions all over the place now’ (id 19 SW)
Reasons for ultimately having the vaccination despite being initially sceptical	‘the vaccination would have had to have gone through you know, rigorous testing and stuff, so you know, I was I, I had them done with you know, no issues really. I, obviously, did a bit of, you know, um, research stuff, and I, I, and I spoke to a few people like um, my GP and stuff, about vaccinations, to get some reassurance’. (id 16 SW) ‘I've just sort of accepted it, because you know it's, it's the best thing to do, and you've been told it's the best thing to do, whether or not it is, you just don't know, you know, I don't think anybody can give an accurate answer yet’ (id 13 CW)

### General changes to DCW roles during the pandemic

DCWs reported several changes to their roles since the start of the pandemic, including an increased workload, greater use of PPE, using rapid antigen test kits (referred to here as lateral flow testing [LFT] kits), organisational changes to clients' homes and offices and greater use of technology and virtual communication (described in more detail in Table 2). To note. At the time of the interviews, the PPE requirements for DCWs in Wales were a fresh clinical face mask/visor/ plastic apron/gloves before each visit.

**TABLE 2:** Changes to DCW roles

Change	Description
Workload	Increased workload due to staff shortages
Increased safety management precautions in clients' homes	Increased use of and disposal of PPE, domestic cleaning, the cancellation of external outside cleaning agencies, reduced time spent at client calls, and reduced client social activities
Testing	LFT, temperature checking for both DCWs and clients, and logging of results. Arranging PCR tests
Prioritisation of calls for clients most in need	Including increased emotional care and shopping for clients normally reliant on families and friends
Education	Checking clients' understanding of safety measures and choosing specific DCWs to take on COVID‐19 focus
Organisation of client homes	Improved safety focus on clients recently discharged from hospital, reorganisation of supported living houses workspace and dedicated staff to separate caring for clients with COVID‐19 symptoms
Staff reorganisation	Movement of staff to other supported living houses to manage staff shortages
Organisational changes to DCW offices	Reorganisation of furniture and increased cleaning to promote safety of office‐based care workers and those picking up provisions
Use of virtual communication	To aid within team contact and clients/family contact
Shielding	DCWs considered high risk by their General Practitioner advised to shield and not attend work
Isolating	DCWs with symptoms of COVID‐19 were required to go home, self‐isolate and take a PCR test. DCWs required to isolate if in contact with someone who had tested positive

Abbreviations: LFT, lateral flow (rapid antigen) testing kits; PCR, polymerase chain reaction.

### Practical challenges for DCWs in the workplace

#### Staff shortages

Staff shortages increased significantly during the pandemic (Box 1). Staff had been advised to shield if considered vulnerable, while others had to isolate, stop working and wait for the results of PCR tests. Some staff remained at home to look after their children since usual carers such as grandparents needed to shield. Some staff were fearful of coming to work during the pandemic, and participants suggested that fear was also the reason for fewer DCW job applications. Participants discussed that media coverage hindered recruitment through anxiety‐provoking messages that were considered misleading. Participants indicated that new recruits did not have the required personal qualities for the role. Staff in post had to take on the extra work, including those DCWs with an advanced role, they were busier as hospitals discharged many clients back into the community for their safety.

#### Clients/families and private workers not following safety procedures

Several DCWs (mostly care workers) stated that some families of clients did not follow the COVID‐19 precautionary rules, especially wearing face masks. In these instances, DCWs were required to report this behaviour to social workers who would ring the families to remind them that the client was vulnerable. DCWs would also need to increase cleaning and ask families not to visit their relatives when the DCWs were present. Some clients also appeared not to follow safety precautions, for example, vulnerable clients visiting friends' houses or talking to service employees such as gardeners. Some clients became cross when challenged, with some DCWs not having had any guidance on handling such a situation resulting in uncertainty of the appropriate response. It was also reported that some private care workers did not wear face masks around the clients (one reportedly stating that there was no need as they had previously been infected with COVID‐19).

#### Initial shortages of PPE

Several participants reported shortages of PPE relating to face masks and PPE required for working sleepover night shifts such as plastic mattress covers. One carer reported that a friend even started making masks for them.

#### DCW criticism of standard‐issue PPE

The efficacy and comfort of standard PPE issued to DCWs were questioned with reports of visors steaming up when bathing clients resulting in frequent cleaning or raising of visors to ensure visibility. Some DCWs bought PPE which they considered were of a superior standard claiming it stopped them from acquiring COVID‐19. Some visors were described as looking like a welding mask. Another criticism of PPE was the time required to put these on.

#### Client difficulty with PPE

Several DCWs reported clients having problems with them wearing face masks. For example, clients who were hard of hearing or would usually lip‐read could not understand the carer. Ways of dealing with this included speaking louder, using visors instead of face masks, or following supervisor advice to stand away from the client and pull down their face mask to speak. Some clients who could not recognise the DCWs in full PPE would try to pull their face masks off. Some support workers used the language programme Makaton (2022), which utilises signs, speech and symbols to improve communication and some described how they drew faces and painted smiles on the face coverings if clients felt frightened or intimidated. Although many DCWs stated that they would never pull down their face masks and would instead speak louder, gesture and use facial expressions, one support worker said that they would pull down their face mask briefly to smile at the clients while standing away from them. Other DCWs said they would take time to explain to the clients why they were wearing PPE while showing others wearing them on television, which they said helped. One client was annoyed with the DCW wearing gloves and told them to take all the PPE off.

#### Management of lateral flow testing

Although LFT kits had been supplied to many DCWs, some were still waiting for these at the time of the interview (February 2021). Many DCWs reported that staff used these twice weekly for asymptomatic testing. The majority of DCWs were positive about using LFTs, which provided peace of mind that they did not have a COVID‐19 infection to transfer to clients or family members. Nevertheless, some DCWs said the tests came too late, stating the hospitals and care homes received testing kits before DCWs did. They stated that this resulted in staff taking sick leave if symptomatic, which caused staff shortages. Some DCWs queried the efficacy of LFTs proposing that they deliver false positives.

### Lack of Governmental/Employer Preparation for a Pandemic

#### Reorganisation of staff, clients and services

During lockdown, participants reported that hospitals were discharging clients back into the community to safeguard them against hospital‐acquired COVID‐19. This caused alarm amongst several DCWs and the clients, for example a client tested negative on discharge from the hospital, however, tested positive when they were at home. Respite houses accommodate those requiring care, enabling short‐term breaks for caregivers, however during the pandemic, many of these respite houses closed, with only emergency respite houses remaining open. Some support workers were redeployed to existing supported living homes that were experiencing staff shortages. The requirement for support workers to visit several homes created anxiety especially prior to the greater availability of LFTs. The transition from support worker to a care worker role due to care worker staff shortages was reported as being very difficult, for example, insufficient time to become acquainted with the clients and finding their homes (Box 2).

#### Inadequate or confusing information for DCWs

Sources of information sought by participants regarding COVID‐19 included government, local authorities, public health organisations, mainstream media websites, television news, social media and newspapers. Employers provided information to DCWs via managers, support telephone lines, newsletters, blogs, emails, leaflets and posters. WhatsApp support groups were set up to enable DCWs to stay in contact with each other. However, several DCWs, particularly support workers were unhappy with this information. For example, participants noted that the employer information was the same information that was being reported on television. It was proposed that clearer guidance was required from management since information appeared contradictory and changed a great deal. Some felt information from employers was inadequate, resulting in DCWs checking Government and NHS sites for updates on changes, for example on PPE use. Some information was considered contradictory because the Welsh Government's advice was different from that being offered in other UK countries.

#### COVID‐19 training and the need for improved practical instruction

A minority of DCWs stated that they had received no training specifically related to COVID‐19. For those who had received training, most had undergone online training which included the use of professional trainers, videos and assessments. Only one support worker reported receiving face‐to‐face training. DCWs were unsure of the training source but it was suggested to be NHS, government, local authority based or from their Human Resources department. Several DCWs reported the use of the UK‐based online training Social Care TV (2022) which offers e‐learning for health and social care providers. One care worker with an advanced role made videos for DCWs regarding PPE use. The content of this training mostly focused on wearing PPE, being aware of COVID‐19 infection symptoms and how to practice safely. Later during the year, training also consisted of using LFT kits. Some DCWs noted however that the training that they had received needed to be more specific to their work role, for example, how to manage situations when their glasses were steaming up whilst wearing a face mask, when to wear face masks when eating and the acceptability of handwashing using the client's kitchen sink. Because of the lack of knowledge regarding the management of COVID‐19 in actual practice, participants described how DCWs discussed these issues amongst themselves. Training suggestions included involving clients in educational videos rather than actors and using real‐life scenarios, for example when trying to care for clients who disliked PPE. Furthermore, it was reported that much of the training was focused on regulations in England which were different to those in Wales.

#### Limited official standard risk assessments

Participants described that staff returning to work after being advised to shield underwent a risk assessment. However, only one DCW (in an advanced role), was aware of the Welsh Government Risk Assessment Tool (Welsh Government, 2021). DCWs stated that risk assessment tools were usually used by the employer managers and had little knowledge of these tools themselves. Examples of assessments included those used by the health and safety team and a template that was sent to them from the central management team. External companies were also used to assess staff who were deemed to be at a higher risk. Many risks assessments were, however, informal with participants reporting that they assessed themselves or each other. Indeed, one participant reported that the company did not know that they had mild asthma. Some DCWs reported that they had to read guidance on risk assessment and sign that they had done so, others stated that they were unaware of any risk assessments for DCWs.

### Pressure to attend work during the pandemic

Many DCWs were thankful for the statutory sickness payment enhancement scheme made available by the Wales Government (Welsh Government, 2020). However, this was perceived by some as being too low. Several DCWs reported that they had casual contracts or zero‐hour contracts where an employer is not obliged to ensure a minimum number of working hours. Consequently, participants reported they would not get paid if they missed work due to sickness or self‐isolating. Others reported that they did not earn enough money to qualify for sickness payment which would not include hours accrued working overtime while others still stated that some contracts stipulated that sickness payment would not start for 3 days. It was proposed that some DCWs who worked zero‐hour contracts may not be able to afford to take a reduction in pay and may therefore be reluctant to report symptoms of COVID‐19 or may return to work too soon. One DCW said that they actually knew people who had gone into work with symptoms. It was also stated that it was unethical that DCWs should lose money for being honest by taking a test for COVID‐19. Some DCWs were given the option of taking annual leave instead to avoid losing pay. Others were told that if they wanted to continue their role as a DCW, they must give up any additional employment that they had elsewhere, and this also caused a reduction in pay. As well as financial pressures to attend work, the DCWs mentioned feelings of guilt for taking sick leave due to COVID‐19 because they knew that their work colleagues were understaffed. One DCW reported that it was considered unacceptable amongst their work colleagues not to work a scheduled weekend and an example was given of a DCW completing their weekend shift despite feeling ill. A minority of DCWs felt that management had not been adequately supportive during the pandemic causing them to feel pressure to attend work. One stated that it was management who decided if they had COVID‐19 symptoms, rather than sending them for a test. Conversely, a DCW in an advanced role believed that some staff were being untruthful about having COVID‐19 symptoms making it difficult for them to alter rotas (Box 3).

### Perceptions of COVID‐19 risk and of vaccinations

#### Perceptions of becoming infected with the COVID‐19 Virus

Whilst some DCWs stated that they were still being careful to wear full PPE after being vaccinated, a minority stated that they were not worried about getting the virus, for example if living in areas with low COVID‐19 case rates. It was proposed too that client families were becoming complacent with some no longer wearing face masks, with one example being because it was unnecessary as they had previously been infected with the virus. Post‐vaccination complacency was also proposed amongst the DCWs with reports that some were not adhering to the 2‐m distancing rule when taking off PPE outside of a clients' house. One DCW held the view that the virus had been manufactured to kill the older generation but had failed in doing so as many young people had also died (Box 4).

#### Perceived efficacy of COVID‐19 Vaccination

The vast majority of DCWs trusted in the vaccinations and at the time of interview had either received two doses of vaccinations or were waiting for them. Participants reported benefits included that they would help millions of people, it would protect the DCWs from catching COVID‐19 and would protect their clients as they would no longer transmit the virus. It was stated that having had the vaccinations made the clients feel safer. Some stated that they were pleased that their employer was supportive of vaccinations for them. It was asserted that vaccination should be a priority, that proof of vaccination is no different to showing a driving licence or a passport to travel and those yearly vaccinations should not be a problem. It was proposed that COVID‐19 vaccination was more important than influenza vaccination since COVID‐19 was more serious. One DCW stated that they had received the COVID‐19 and influenza vaccinations as they did not want to suffer from both viruses simultaneously. The main reasons for being reluctant to have the COVID‐19 vaccination was insufficient information about its current and long‐term safety profile and that it could be dangerous and kill people especially since adverse events such as blood clots had been reported globally. A minority of the DCWs queried the safety of the vaccination composition proposing, for example, that it included poisonous heavy metals, another participant believed that a microchip was being inserted during vaccination. Fertility issues was a concern which led to one DCW refusing vaccination. Indeed, it was stated that at the onset of the pandemic, public health officials had advised pregnant women not to have the vaccine. Other reasons included the vaccine not preventing illness and the futility of having a vaccination for a virus which mutates leading to the vaccine becoming ineffective, suggesting natural immunity was a better option. Several DCWs felt that they had no option but to have the vaccination as they did not want to lose their jobs and wanted to get on with their lives. Moreover, it was proposed that the virus would be less severe if they had been vaccinated with one DCW deciding to have the vaccination as they knew someone who had become extremely ill with the virus. Some had researched the safety profile of the vaccination themselves and had spoken to others including their GP. It was asserted however that even though some DCWs had been told by their manager that they had the option of having the vaccination, they were subsequently told that they would all be vaccinated. Others still refused though with one stating that they would only be vaccinated if it were necessary to go abroad on holidays.

## DISCUSSION

We interviewed 24 registered DCWs actively working for at least 4 months during one or both initial waves of the COVID‐19 pandemic in Wales. Interviews explored experiences and working practices during the pandemic and factors that may increase risks of COVID‐19 infection. Key themes emerging from the interviews included changes to DCW roles, workplace challenges for DCWs, lack of pandemic preparation, pressures to go to work and perceptions of COVID‐19 risk and of vaccinations.

Several studies have described DCWs risks of infection whilst working during the COVID‐19 pandemic, for example in England (Nyashanu et al., 2020a, 2020b) and in the US (Sterling et al., 2020). This is the first paper to focus on risks relating to the DCW role.

A lack of pandemic preparedness was reported in our study and by Nyashanu et al. (2020b) whose care workers reported no clear strategic policy regarding the pandemic. The practice of discharging people from hospital back to the community for their safety is problematic because of the risk of hospital‐acquired COVID‐19 (Healthcare Safety Investigation Branch, 2020; Hodgson et al., 2020; Oliver, 2021). As in our data, Nyashanu et al. (2020a) too reported that care workers felt under pressure to accept hospital patients when no testing was available. Moreover, Sterling et al. (2020) reported that care workers had to work with new clients as some of the existing clients cancelled home care services for fear of catching COVID‐19.

Working in close proximity with clients is an established feature of the DCW role and for which mitigation through PPE use is already part of the role albeit much less in pre‐pandemic times. However, the initial lack of PPE during the pandemic was noted by several other studies (Nyashanu et al., 2020b; Sterling et al. 2020) with care workers reporting the purchase of their own supplies or reliance on family and friends for supplies (Sterling et al., 2020). Nyashanu et al. (2020b) highlighted that the PPE was deemed not fit for purpose. Our study also described the impracticalities of clients often disliking PPE and that care workers found them unsuitable for working with clients, sometimes resulting in face masks being removed. Although LFTs eventually became available, providing reassurance to DCWs in our study, some questioned their efficacy as have other care workers (Nyashanu et al. 2020b). Moreover, the limited use of the official Welsh Government COVID‐19 risk assessment tool (2021) and instead the informal risk assessments reported in our study may have disadvantaged many DCWs who were unaware of heightened occupational risks. Sterling et al. (2020) reported that care workers had a lack of specific training although our study was more precise indicating that training needed to be more tailored to a DCW's role.

Increased staff shortages were not foreseen in spite of them already being an existing challenge with care workers (Age UK, 2020). This issue, reported in other studies, for example, that fear of infection caused sickness rates to increase with some staff taking annual leave entitlement (Nyashanu et al., 2020a) and others still reporting that self‐isolation and delay in testing resulted in lack of staff (Nyashanu et al., 2020b). In spite of the fear of being infected with COVID‐19 whilst at work, financial pressures of those on contracts with no sickness benefits may have pressured DCWs to work. These contracts have been noted as problematic in the UK before the pandemic (Ravalier et al., 2019) but have still not been resolved. Furthermore, Sterling et al. (2020) stated that US care workers had to balance facing the risk between their own health and their financial wellbeing. Our study though highlighted that DCWs might actually work in spite of having COVID‐19 symptoms for fear of losing pay.

The inadequacy of information during the pandemic noted by our data was reflected in other studies too. Sterling et al. (2020) reported care workers relied on sources including the news and social media and Nyashanu et al. (2020a, 2020b) highlighted that care workers did not receive any specific working guidance from central government and that there were many changes to guidance. A lack of information could also be the reason for vaccination hesitancy in DCWs.

We have built on the emergent themes to summarise key areas of risk either in terms of exposure to COVID‐19 infection or to other adverse health outcomes (Appendix S1.). For challenges identified, we recommend mitigation strategies. In several instances, a common response option may address several risks. Two examples are the recommendations for enhanced training for care workers and for teamwork support. It is perhaps unsurprising that various new learning needs are evident (e.g. related to PPE, testing, vaccination) and a common approach to addressing that would seem efficient. Similarly, the pandemic placed stress on a sector with existing workforce challenges and some common responses such as models to enhance teamworking, particularly when working in relative isolation from other members of the workforce would seem appropriate.

We aimed to set the challenges and recommendations (Appendix S1.) within a broader socio‐ecological framework (Ingram et al., 2021) via the levels of response to emphasise the layered and interactive nature of the challenges experienced by DCWs. Governments make policy and critical, funding decisions that create the broad context within which services to clients are provided. This includes pre‐pandemic funding overall to the sector as well as policy responses such as purchasing PPE, the funding and rolling out of vaccination. However, government policies need to be enacted by provider organisations, who will also be responsible for management decisions affecting service provision. Our interviews highlighted key features of the interpersonal context such as the presence and behaviour of the client family members, friends and other on‐site staff. Finally, the actions of DCWs driven by their own experiences, understanding and perspectives play an essential role in managing risks. Laying out the risks and recommendations within this framework highlights how effective action can only really be designed and implemented by addressing the multiple influences on practice.

The qualitative study adds strength to the overall OSCAR study by providing contemporary carer‐reported insights into risk factors for COVID‐19 exposure that can inform the quantitative modelling (Lugg‐Widger et al., 2021). Of even greater value perhaps is the detailed nuance achieved about how some risk factors and outcomes are experienced in practice and how interventions to address them may be introduced or improved. While the quantitative analyses will explore mental health outcomes for care workers before and during the pandemic, this qualitative study details a wide range of contributing factors such as workload, financial pressures and uncertainties about protective measures such as PPE availability, use and the role of vaccines.

Efforts were made to maximise the diversity of care workers interviewed by monitoring the socio‐demographic profile of the emerging interviewee sample and exploring a range of advertisement and recruitment routes. Although recruitment was concluded at the point where no new major themes were emerging from the interviews, the sample is still likely to reflect bias and it is plausible that different issues may have been reported from a larger and even more diverse group of DCWs. However, the study was not expected to enable fully generalisable findings and it is likely that most broad concerns have been identified, something that appears to be borne out by both contemporarily reported findings and the views of our stakeholder group (Lugg‐Widger et al., 2021).

Nevertheless, further questions worth exploring include a more detailed examination of how organisations continue to implement risk assessments and DCW experiences and responses to the initial vaccine rollout and subsequent booster programme. For the former, gaining insights from an organisational perspective may be particularly useful, in addition to those of the DCWs themselves. For the latter, tracking emerging views on vaccination against COVID‐19 will be especially important in the context of mandatory vaccination for social care staff in client‐facing roles and ongoing workforce pressures (UK Government, 2021).

## CONCLUSIONS

DCWs described a range of experiences that may have served to increase the risk of exposure to COVID‐19. These risks operate at varying levels of the socio‐ecological context within which care workers operate and recommended courses of action to address them may similarly be required at different levels and by different actors. In‐depth understanding of the nature of risk factors can both inform health outcome analyses and interpretation, as well as guide responses to reduce risk including through optimisation of existing approaches such as increasingly tailored training.

## AUTHOR CONTRIBUTIONS

MR and RCJ are the chief investigators. HP conducted the interviews, LBH was the second coder. FLW contributed to the recruitment and ethical approval of study documents. HP & FLW drafted the paper. All authors contributed to the design and analysis and advised on the drafts of the manuscript. All authors read and approved the final manuscript.

## FUNDING INFORMATION

Research Councils UK., Economic and Social Research Council‐ES/V015206/1.

## CONFLICT OF INTEREST

All other authors declare that they have no competing interests.

## Supporting information


Appendix S1.

Appendix S2.

## Data Availability

Data supporting this study will be available in UK Data Service data repository ReShare https://reshare.ukdataservice.ac.uk/

## References

[hsc14109-bib-0001] Age UK (2020) The impact of Brexit on care. https://www.ageuk.org.uk/our‐impact/campaigning/care‐in‐crisis/brexit/

[hsc14109-bib-0002] Braun, V. , & Clarke, V. (2006). Using thematic analysis in psychology. Qualitative Research in Psychology, 3, 77–101. 10.1191/1478088706qp063oa

[hsc14109-bib-0003] Hayes, L. , Tarrant, A. , & Walters, H. (2020). Care and support workers' perceptions of health and safety issues in social care during the COVID‐19 pandemic: Initial findings. https://media.www.kent.ac.uk/se/11148/CareworkersHealthandSafetyreport15042.pdf

[hsc14109-bib-0004] Healthcare and Safety Investigation Branch . (2020). COVID‐19 transmission in hospitals: management of the risk—A prospective safety investigation. https://www.hsib.org.uk/investigations‐and‐reports/covid‐19‐transmission‐in‐hospitals‐management‐of‐the‐risk/

[hsc14109-bib-0005] Hodgson, K. , Grimm, F. , Vestesson, E. , Brine, R. , & Deeny, S. (2020). Briefing: Adult social care and COVID‐19. Assessing the impact on social care users and staff in England so far. Health Foundation. https://www.health.org.uk/publications/reports/adult‐social‐care‐and‐covid‐19‐assessing‐the‐impact‐on‐social‐care‐users‐and‐staff‐in‐england‐so‐far

[hsc14109-bib-0006] Ingram, M. , Wolf, A. M. A. , López‐Gálvez, N. I. , Griffin, S. C. , & Beamer, P. I. (2021). Proposing a social ecological approach to address disparities in occupational exposures and health for low‐wage and minority workers employed in small businesses. Journal of Exposure Science & Environmental Epidemiology, 31(3), 404–411. 33774651 10.1038/s41370-021-00317-5 PMC8003897

[hsc14109-bib-0024] Lugg‐Widger, F. V. , Cannings‐John, R. , Akbari, A. , Brookes‐Howell, L. , Hood, K. , John, A. , Jones, H. , Prout, H. , Schoenbuchner, S. , Thomas, D. , & Robling, M. (2021). Establishing the impact of COVID‐19 on the health outcomes of domiciliary care workers in Wales using routine data: a protocol for the OSCAR study. International Journal of Population Data Science, 5(4). 10.23889/ijpds.v5i4.1656 PMC8280712 34345715

[hsc14109-bib-0007] Makaton . (2022). https://makaton.org/

[hsc14109-bib-0008] Nyashanu, M. , Pfende, F. , & Ekpenyong, M. (2020b). Exploring the challenges faced by frontline workers in health and social care amid the COVID‐19 pandemic: Experiences of frontline workers in the English midlands region, UK. Journal of Interprofessional Care, 34(5), 655–661. 32674701 10.1080/13561820.2020.1792425

[hsc14109-bib-0009] Nyashanu, M. , Pfende, F. , & Ekpenyong, M. S. (2020a). Triggers of mental health problems among frontline healthcare workers during the COVID‐19 pandemic in private care homes and domiciliary care agencies: Lived experiences of care workers in the midlands region. Health & Social Care in the Community. 10.1111/hsc.13204 33107131

[hsc14109-bib-0010] Office for National Statistics . (2020). Coronavirus (COVID‐19) related deaths by occupation, England and Wales. https://www.ons.gov.uk/peoplepopulationandcommunity/healthandsocialcare/causesofdeath/bulletins/coronaviruscovid19relateddeathsbyoccupationenglandandwales/deathsregisteredbetween9marchand25may2020

[hsc14109-bib-0011] Oliver, D. (2021). Could we do better on hospital acquired covid‐19 in a future wave? BMJ, 372, n70. 10.1136/bmj.n70 33441317

[hsc14109-bib-0012] Public Health England . (2020). Pilot point prevalence survey of COVID‐19 among domiciliary care staff in England. https://www.gov.uk/government/publications/covid‐19‐prevalence‐survey‐domiciliary‐care‐staff‐in‐england

[hsc14109-bib-0013] QSR International Pty Ltd . (2018). NVivo (Version 12), https://www.qsrinternational.com/nvivo‐qualitative‐data‐analysis‐software/home

[hsc14109-bib-0014] Ravalier, J. , Morton, R. , Russell, L. , & Rei Fidalgo, A. (2019). Zero‐hour contracts and stress in UK domiciliary care workers. Health & Social Care in the Community, 27(2), 348–355. 30175441 10.1111/hsc.12652

[hsc14109-bib-0015] Skills for Care . (2021). The size and structure of the adult social care sector and workforce in England. https://www.skillsforcare.org.uk/adult‐social‐care‐workforce‐data/Workforce‐intelligence/publications/national‐information/The‐size‐and‐structure‐of‐the‐adult‐social‐care‐sector‐and‐workforce‐in‐England.aspx

[hsc14109-bib-0016] Social Care TV . (2022). https://www.social‐care.tv/?gclid=EAIaIQobChMIqPvV9rOx9QIVgdd3Ch1Q1Q‐wEAAYASAAEgJgf_D_BwE

[hsc14109-bib-0017] Social Care Wales . (2018). The domiciliary Care worker: Practice guidance for domiciliary care workers registered with Social Care Wales. https://socialcare.wales/cms_assets/file‐uploads/Practice‐Guidance‐Version‐1.pdf

[hsc14109-bib-0018] Social Care Wales . (2021a). Domiciliary care workers on the register – 1 April 2021. https://socialcare.wales/cms_assets/file‐uploads/Domiciliary‐care‐worker‐ENG.pdf

[hsc14109-bib-0019] Social Care Wales . (2021b). Registration: Why we register. https://socialcare.wales/registration/why‐we‐register

[hsc14109-bib-0020] Sterling, M. R. , Tseng, E. , Poon, A. , Cho, J. , Avgar, A. C. , Kern, L. M. , Ankuda, C. K. , & Dell, N. (2020). Experiences of home health care workers in new York City during the coronavirus disease 2019 pandemic: A qualitative analysis. JAMA Internal Medicine, 180(11), 1453–1459. 32749450 10.1001/jamainternmed.2020.3930 PMC7404061

[hsc14109-bib-0021] UK Government . (2021). Government to introduce COVID‐19 vaccination as a condition of deployment for all frontline health and social care workers. https://www.gov.uk/government/news/government‐to‐introduce‐covid‐19‐vaccination‐as‐a‐condition‐of‐deployment‐for‐all‐frontline‐health‐and‐social‐care‐workers

[hsc14109-bib-0022] Welsh Government . (2020). COVID‐19 statutory sick pay enhancement scheme. https://gov.wales/covid‐19‐statutory‐sick‐pay‐enhancement‐scheme

[hsc14109-bib-0023] Welsh Government . (2021). All Wales COVID‐19 workforce risk assessment tool 2021 https://gov.wales/covid‐19‐workforce‐risk‐assessment‐tool

