# Supplementary material for: “I don't mean to be rude, but could you put a mask on while I'm here?” A qualitative study of risks experienced by domiciliary care workers in Wales during the COVID‐19 pandemic
Source: Health Soc Care Community. 2022 Nov 24;30(6):e6601–12. doi: 10.1111/hsc.14109 (PMC10100139; doi:10.1111/hsc.14109)
Supplement: Supplementary file 1 — Appendix S1. [file HSC-30-e6601-s001.docx]

Appendix 1. Recommendations

The OSCAR study team is based in the Centre for Trials Research, Cardiff University. Our interviews with Domiciliary Care Workers (DCWs) about their experiences of providing care during the COVID-19 pandemic identified several challenges. We summarise these findings, which contribute to the risk of e*xposure to COVID-19,* or to other adverse outcomes such as *emotional or wellbeing burden.* Our recommendations identify different points at which intervention may be helpful, for example at policy or organisational level.
The need for tailored training is also identified to further address some challenges for example, on how to manage client and family expectation.

| *Summary of challenges* | | *Level of Response* | | | | | Recommendations | *Tailored Training* |
| --- | --- | --- | --- | --- | --- | --- | --- | --- |
|  |  | **Policy** | **Community** | **Organisational** | **Interpersonal^1^** | **Personal** |  |  |
| *Risk of exposure to COVID-19* | ***Lack of domiciliary care workers:*** Staff shortages due to shielding and sickness as well as ongoing staff shortages. | ✓ |  |  |  |  | Funding to support sector to address sickness, recruitment / retention and increase in demand of services. |  |
|  | ***Providing personal client care:*** Core work involves personal / proximal care to client in their own home.^2^ | ✓ |  | ✓ |  | ✓ | Maintain funding and adequate stocks of PPE, Lateral Flow Tests.  Evidence based changes on requirement for PPE to be communicated by policy clearly and rapidly to employees and DCWs. | ✓ |
|  | ***Lack of PPE:*** Initial lack of Personal Protective Equipment (PPE) experienced by some DCWs. Infection risk without PPE. | ✓ |  | ✓ |  |  |  |  |
|  | ***Unknown COVID-19 status:*** COVID-19 status of both DCWs and clients unknown causing uncertainties relating to infection risk. | ✓ |  | ✓ |  |  |  |  |
|  | ***Patient discharge from hospital:*** Discharge of hospital patients of unknown COVID-19 status. | ✓ |  | ✓ |  |  | Effective discharge screening and planning. |  |
|  | ***Lack of formal risk assessments:*** Lack of formal risk assessments by employers resulting in some DCWs assessing each other for COVID-19 risk. Possible inadequate assessment and mitigation. | ✓ |  | ✓ |  | ✓ | Develop and implement COVID-19 risk assessment for DCWs that accounts for inherent risks of role. |  |
|  | ***Clients/families not following safety guidance:*** Clients and families not adhering to COVID-19 safety guidance (e.g. wearing masks and household mixing, risking infection). | ✓ | ✓ | ✓ | ✓ | ✓ | Pro-active and clear signposting of trusted information and guidance to DCWs and their clients on safety, efficacy and management of COVID-19 prevention measures.  DCWs, clients and families to access information.  Pro-active conversations with DCWs in regions with higher hesitancy by public health officials. | ✓ |
|  | ***Efficacy of lateral flow tests queried:*** DCWs querying efficacy of LFTs, possibly leading to non- / inconsistent use. | ✓ |  | ✓ |  | ✓ |  |  |
|  | ***Perceptions of lower risk post vaccination:*** DCWs felt less at risk / lack awareness of catching and transferring COVID-19 post vaccination. | ✓ |  | ✓ |  | ✓ |  |  |
|  | ***Vaccination hesitancy:*** Lack of information about the vaccination including safety concerns, leading to reduced vaccination uptake. | ✓ | ✓ | ✓ |  | ✓ |  |  |
|  | ***UK wide COVID-19 guidance confusing***: Real and apparent differences between Wales and England (or UK) policies shared on mainstream media. This could lead to using incorrect policies or ‘policy overload’. | ✓ |  | ✓ |  |  |  |  |
|  | ***PPE not practical with clients:*** Clients’ fear / dislike of PPE (e.g. unable to hear DCW due to reduced hearing) potentially leading to improper mask use. |  |  | ✓ | ✓ | ✓ |  | ✓ |
|  | ***COVID-19 training not tailored to role:*** COVID-19 training not focused on DCW practical tasks resulting in difficulties, uncertainties and improper use (e.g. glasses steaming up when bathing clients when using standard masks). | ✓ |  | ✓ |  |  | Review current procedures and engage with DCWs on their views (choice of PPE, role descriptions, contracts and sickness payments).  Risk assess those DCWs whose role changes for increased risk of COVID-19 infection.  Continued investment, including of the COVID-19 statutory sick pay enhancement scheme in Wales. | ✓ |
|  | ***Reassignment of carer roles:*** DCW reassigned to several supported living houses following closure of respite houses opening up risk of COVID-19 infection to DCWs and clients. |  |  | ✓ |  |  |  | ✓ |
| *Emotional or wellbeing burden* | ***Sickness payments and contracts:*** Pressure to attend work despite illness/COVID-19 status and stress due to no sickness payment. | ✓ |  | ✓ |  |  |  |  |
|  | ***Isolation and loneliness:*** Working patterns driven by pandemic restrictions causing loneliness and isolation. |  |  | ✓ |  | ✓ | Enhanced teamwork, good news sharing and improved communication between DCW teams - Employer and staff engagement via social media and communication platforms.  Increased signposting to support. Acknowledgement of challenges by employers. national media, political leaders. |  |
|  | ***Fear of COVID-19:*** For self, families and clients causing anxiety. |  |  | ✓ |  | ✓ |  |  |
|  | ***Feeling under-valued:*** Lack of recognition of value in job role. | ✓ | ✓ | ✓ |  | ✓ |  |  |
|  | ***Increased workload for staff in an advanced role:*** DCWs in mainly office-based role (Team Leader) now with / greater client contact increasing workload & stress. |  |  | ✓ |  |  |  |  |
|  | ***Lack of support from employers:*** Some employers perceived as unfamiliar with practical working conditions, sceptical of worker symptoms - leading to low worker morale. |  |  | ✓ |  |  |  |  |
| **^1^Interpersonal definition:** DCW interactions with those around them, in this context including clients, colleagues, families and friends.  ^2^ Although providing personal client care is a core feature of the DCW role, here we focus on additional mitigation strategies. | | | | | | | | |

Formulating recommendations based on problems emerging from the interviews involved sharing information with the study team’s Implementation Reference Group. The group comprises stakeholders from across the UK and representing a range of organisations (e.g. government, skills councils, regulators, service providers, trades union) as well as care workers and a client. The group’s input provided a policy and practice perspective on the problems encountered and allowed the shaping of context-relevant recommendations. It should also be noted that problems identified included those that may either be largely un-addressable (e.g. proximal client working is essential to care provision), less easy to immediately address (e.g. increasing and then benefitting from enhanced governmental funding to the care sector), already partially addressed (e.g. introduction of pandemic related guidance) and where existing responses could be further optimised (e.g. greater specificity in existing training content, ensuring widespread implementation of supportive strategies to DCWs)
