# Supplementary material for: “I don't mean to be rude, but could you put a mask on while I'm here?” A qualitative study of risks experienced by domiciliary care workers in Wales during the COVID‐19 pandemic
Source: Health Soc Care Community. 2022 Nov 24;30(6):e6601–12. doi: 10.1111/hsc.14109 (PMC10100139; doi:10.1111/hsc.14109)
Supplement: Supplementary file 2 — Appendix S2. [file HSC-30-e6601-s002.docx]

Appendix 2. Topic Guide

Interview topics V1.4 09.02.21

Can you tell me a little bit about how long you have been working as a DCW and what you do in this role?

- Has your work changed since the COVID-19 outbreak?
- Has your employer made any changes to your work role?
- What have you found to be helpful or least helpful?
- Have you undertaken any additional risk assessment for your role?
- Have you received any training and/or mentoring since the COVID-19 pandemic?
- Has the COVID-19 pandemic affected you personally in the way you do your job?
- What do you think of testing for COVID-19 and testing for COVID-19 with no symptoms?
- Will you be having theCOVID-19 jab and what are your thoughts on this jab?
- Do you usually have the flu jab and what are your thoughts on vaccinations in general?
- Is there anything else you would like to say about your work role and COVID-19?
